# Optimizing the Composition of Solid Sodium Silicate-Activated Solid Waste-Based Geopolymer Based on the Response Surface Methodology and Its Performance

**DOI:** 10.3390/ma19071438

**Published:** 2026-04-03

**Authors:** Huiyong Zhou, Yanchao Wang, Hua Gao, Wei Guo, Taotao Fan, Chundi Si, Xibao Ma

**Affiliations:** 1 Hebei Shi-Tai Expressway Development Co., Ltd., Shijiazhuang 050299, China; 13231911282@163.com (H.Z.); 13462924524@163.com (Y.W.); 15690101837@163.com (H.G.); 2 School of Traffic and Transportation, Shijiazhuang Tiedao University, Shijiazhuang 050043, China; 19932792304@163.com (W.G.); sichundi@stdu.edu.cn (C.S.); 19732903108@163.com (X.M.); 3 Hebei Key Laboratory of Traffic Safety and Control, Shijiazhuang 050043, China

**Keywords:** alkali-activated geopolymers, industrial solid wastes, solid sodium silicate, response surface methodology, composition optimization, mechanical properties, reaction mechanism

## Abstract

**Highlights:**

- The study employed three solid wastes (slag, iron tailings sand, and coal gangue powder) as precursors to enhance the mechanical properties of geopolymers.
- Application of response surface methodology to optimize the mix design of geopolymers prepared from solid waste mixtures and solid sodium silicate activators, analyzing the effects of slag, coal gangue powder and Na_2_O dosage on geopolymer compressive strength, confirming the positive influence of slag content.
- A multi-faceted durability assessment of the geopolymer revealed its exceptional freeze resistance (mass loss rate of merely 1.67% after 100 freeze–thaw cycles) and salt corrosion resistance (corrosion coefficient >92%).
- Combined with scanning electron microscopy (SEM) testing, the mechanism underpinning its enhanced performance was elucidated at the microscopic level. Within an alkaline environment, the interwoven consolidation of hydrated gels (C-S-H and C-A-S-H) synergizes with the filling effect of unhydrated solid waste, collectively forming a compact structure. This process consequently elevates the mechanical properties and durability of the geopolymer.

**Abstract:**

Alkali-activated solid waste-based geopolymer represents a novel form of inorganic cementitious material, which is one of the key research directions in the building materials field to achieve the targets of carbon peak and carbon neutrality. Therefore, taking solid waste materials as raw materials to prepare the alkali-activated solid waste-based geopolymers with better mechanical properties is of significant importance for expanding the utilization channels of industrial solid waste materials in Hebei Province. In this study, three solid waste materials, slag, iron tailings sand and coal gangue powder, were used as the precursors of geopolymer, and solid sodium silicate was used as the activator to prepare the solid waste-based geopolymer. Response surface methodology was adopted to design the composition of the geopolymer, and the dosages of slag, Na_2_O and coal gangue powder were taken as design variables, and the compressive strength of the geopolymer at 7 days and 28 days were taken as response variables. The results show that it is feasible to optimize the composition of solid sodium silicate-activated solid waste-based geopolymer (SSG) by using response surface methodology. The error value of the SSG-mortar compressive strength prediction model is below 2.0%. The slag contents exhibit a positive correlation with the compressive strength of SSG-mortar, but the coal gangue powder contents and Na_2_O contents have a negative correlation. The optimized compositions of SSG-mortar are 20% iron tailings sand, 26% coal gangue powder, 54% slag, and 6.41% Na_2_O (regulated by 6.23% solid sodium silicate and 6.23% solid NaOH granules), and the corresponding compressive strengths of SSG-mortar at 7 days and 28 days are 37.1 MPa and 44.9 MPa, respectively. In addition, dry shrinkage tests, wet–dry cycling tests, freeze–thaw cycling tests, salt corrosion tests, SEM analysis and XRD analysis were conducted on the SSG-mortar with the optimal composition to evaluate its shrinkage behavior, freeze–thaw resistance, salt corrosion resistance and microstructural strengthening mechanisms. The results show that SSG-mortar has relatively good frost resistance and salt erosion resistance. The mass loss rate value and compressive strength loss rate value of SSG-mortar are 1.67% and 18.7%, respectively, after 100 freeze–thaw cycles. Furthermore, the corrosion resistance coefficient value of SSG-mortar is greater than 92%, and the mass loss rate value is lower than 2.4%. The SEM and XRD test results display that, in an alkaline environment, the interwoven consolidation of hydrated gels (including C-S-H gel, C-A-S-H gel, C-(N)-A-S-H gel and N-A-S-H gel) and the filling effect of solid wastes jointly achieve an improvement in the properties of SSG-mortar.

## 1. Introduction

As an emerging green cementitious material, alkali-activated geopolymer primarily utilizes aluminosilicate solid wastes as the primary raw material. The hydration activity of the raw material of geopolymer is activated by alkaline activators, and a cementitious structure is formed via hydration reactions. Alkali-activated geopolymer exhibits advantages, including high strength, low carbon emissions, and extensive abundant component material sources [1,2,3], and can replace cement in applications such as construction engineering, highway engineering, and backfilling engineering [2,4].

Currently, industrial solid wastes such as iron tailings sand, slag, red mud, coal gangue and fly ash are frequently utilized in the preparation of alkali-activated geopolymers [5,6,7,8]. The types of alkaline activators include solid sodium silicate, liquid sodium silicate, calcium carbide slag, steel slag, alkali slag, sodium hydroxide (NaOH), etc. [9,10,11]. Feng et al. [12] produced alkali-activated geopolymers with favorable mechanical properties, employing coal gangue, fly ash and waste incineration, and NaOH along with solid sodium silicate as activators. Nag et al. [13] synthesized geopolymers via NaOH as the activator in combination with fly ash and fine-grained blast furnace slag. The impact of NaOH concentration on the mechanical characteristics of geopolymers was systematically explored. Wang et al. [14] prepared alkali-activated geopolymers with excellent fluidity, utilizing metakaolin, slag, and marble waste powder of varying particle sizes as solid waste materials, and liquid sodium silicate and NaOH as alkali activators. In conclusion, it is feasible to develop geopolymer by utilizing multi-source industrial solid wastes as raw materials and sodium silicate as alkaline activator. However, the properties of alkali-activated solid waste-based geopolymer are closely linked to the types and proportions of raw materials. When the types of raw materials are limited, determining the optimal composition of geopolymers through scientific design is a key means of ensuring they possess good mechanical properties.

Several scholars researched the composition design of alkali-activated solid waste-based geopolymer. Pattanayak et al. [15] optimized the preparation parameters of geopolymers by integrating artificial neural networks with the response surface methodology (RSM), such as raw material composition, molar concentration of NaOH, curing times, and curing temperature. Aouad et al. [16] designed the composition of geopolymers prepared from kaolin, metakaolin and fly ash by using the simplex centroid design method, and found that, when the blending ratio of metakaolin to fly ash was 25%:75%, the mechanical properties of geopolymer reached an optimal level, namely 20.8 MPa of compressive strength at 28 days. Maataoui et al. [17] applied the central composite design method to explore the optimization of geopolymer preparation parameters. They found that the geopolymer had the best performance, under the conditions of fly ash grinding at 300 rpm for 15 min, 10M solution of NaOH, and curing at 80 °C for 24 h, corresponding to a 28-day compressive strength of 46.47 MPa. Shahmansouri et al. [18] employed the RSM to optimize the mix proportion of pozzolanic-modified slag-based geopolymer concrete. The results showed that optimal mechanical properties would be achieved by substituting 15.9% of slag with natural zeolite combined with 5.28 M sodium hydroxide, or by substituting 30.0% of slag with silica fume combined with 6.19 M sodium hydroxide. Therefore, the mechanical properties of alkali-activated solid waste-based geopolymer are closely related to its composition.

Some studies introduced the durability performance and reaction mechanism of alkali-activated solid waste-based geopolymer. Yang et al. [19] observed that NaOH activated calcium carbide slag-based geopolymers had a good freeze–thaw resistance and salt erosion resistance. Rani et al. [20] found that polypropylene fibers would advance the compressive strength and service life of geopolymer. Sahoo et al. [21] found that, compared with slaked lime and Portland cement, the expansive soil treated with slag-based geopolymers had the properties of excellent strength and durability. Ji et al. [22] assessed the durability properties of municipal solid waste incinerator bottom ash-based geopolymer-stabilized stone, and found that the cumulative dry shrinkage coefficient value of geopolymer-stabilized stone gradually reduced with the increasing dosages of geopolymer. Meanwhile, the strength development of stabilized stone was attributed to the hydration reaction of geopolymer, which generated calcium–silicate–hydrate (C-S-H) gel, sodium–aluminosilicate–hydrate (N-A-S-H) gel and ettringite. Su et al. [23] concluded that the strength formation of slag-fly ash geopolymer-solidified organic clay was mainly related to the degree of clay particle cementation and the content of cementitious products. Yang et al. [24] observed that the addition of slag and fly ash could promote the hydration reaction inside concrete structure, resulting in more C-S-H gel formation and enhancing the strength. The SEM test results reported by Zhang et al. [25] suggested that the main hydration products of geopolymer were calcium–aluminum–hydrate gel and calcium–aluminum–silicate–hydrate (C-A-S-H) gel, and C-S-H gel. The consolidation function of these gels, coupled with the filling effect of non-hydrated geopolymer phases, enhanced the adhesion between geopolymer particles and improved the compressive strength of geopolymer. Therefore, the composition of geopolymer has a significant impact on its durability. Revealing the mechanism of the synergistic effect between different geopolymers phases and their influence on strength formation is critical for advancing high-performance geopolymer.

In summary, although existing research on activated solid waste-based geopolymer has made progress in terms of multi-component raw material composition, the following shortcomings remain: In terms of composition design, the focus has largely been on traditional high-activity solid wastes such as fly ash and slag, whilst research on low-activity solid wastes such as iron tailings sand and coal gangue has been limited. In terms of optimization methods, the determination of optimal mixtures is predominantly based on extensive experimentation. Furthermore, existing studies utilizing response surface methodology typically target a single mechanical property as the optimization objective, with little consideration being given to the synergistic optimization of multiple objectives.

**Figure 1:**
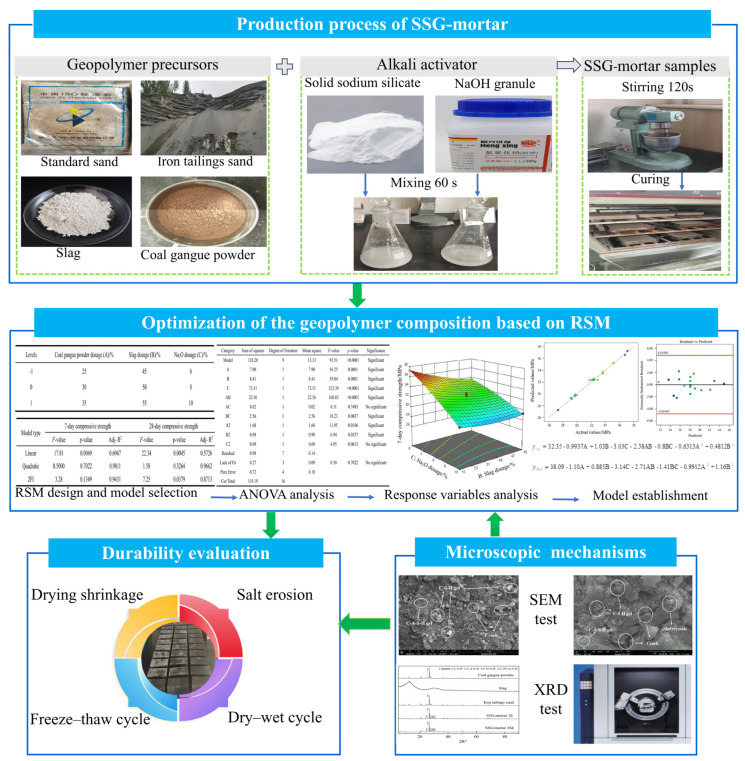
Research framework.

In this study, typical industrial solid wastes from Hebei Province were selected as raw materials, and solid sodium silicate and NaOH were adopted to comprise a composite alkali activation system. The composition ratios of solid sodium silicate-activated solid waste geopolymers (SSGs) were optimized using RSM. The shrinkage behavior, freeze–thaw resistance and salt erosion resistance of SSG-mortar at the optimal mix ratio were evaluated through drying shrinkage, freeze–thaw cycle and salt erosion tests, respectively. The reinforcement mechanism of SSG-mortar was analyzed combined with scanning electron microscopy (SEM) and X-ray diffraction analysis (XRD). The framework of this study is shown in Figure 1.

## 2. Materials and Methods

### 2.1. Raw Materials

#### 2.1.1. Geopolymer Precursors

**Figure 2:**
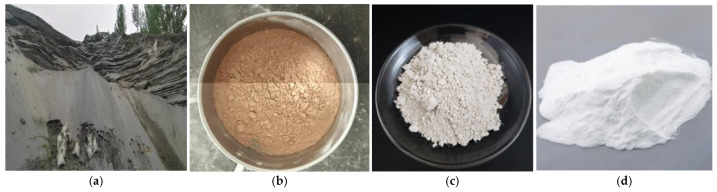
Appearances of raw materials. (**a**) Iron tailings sand. (**b**) Coal gangue powder. (**c**) Slag. (**d**) Solid sodium silicate.

Three industrial solid wastes (namely, iron tailings sand, coal gangue powder and slag) from Shijiazhuang City, Hebei Province were adopted to prepare the geopolymer precursors. The appearances of industrial solid wastes are shown in Figure 2a–c, and their chemical compositions are shown in Table 1. The main components of iron tailings sand are SiO_2_ and Fe_2_O_3_, with a mass percentage of 68.3% and 12.2%, respectively. The main constituents of coal gangue powder are SiO_2_ and Al_2_O_3_, with the mass percentage of 1.5% and 21.0%, respectively. The principal components of slag are CaO, SiO_2_ and Al_2_O_3_, with a total mass percentage of 82.6%. The pozzolanic activity of iron tailings sand and coal gangue powder is relatively low and requires pre-activation treatment. The activation method for iron tailings sand was as follows: mechanical grinding for 60 min and then adding 1% CaSO_4_·2H_2_O. The activation method of coal gangue powder was calcination at 750 °C for 2 h. The activity index values of iron tailings sand and coal gangue powder after activation are 81.50% and 89.28%, respectively.

#### 2.1.2. Alkali Activator

Solid sodium silicate was selected as an alkali activator, as illustrated in Figure 2d. The primary component of solid sodium silicate is Na_2_O·2SiO_2_, which exists as anhydrous silicate crystals with a Na_2_O content of 25.4%. AR96% solid NaOH granules were adopted to adjust the modulus of sodium silicate, while the dosage of alkali activator was calculated by external admixture. The dosage of Na_2_O was adjusted by the amount of solid NaOH granules and solid sodium silicate, which are calculated by Equations (1) and (2), respectively [26].

(1) $$m_{0}=\frac{w_{0}\times 450}{W_{{\mathrm{Na}}_{2}O}+62\times \left(\frac{W_{{\mathrm{SiO}}_{2}}}{60\;\times \;M}-\frac{W_{{\mathrm{Na}}_{2}O}}{62}\right)}$$

(2) $$m_{\mathrm{NaOH}}=\left(\frac{W_{{\mathrm{SiO}}_{2}}}{60\times M}-\frac{W_{{\mathrm{Na}}_{2}O}}{62}\right)\times m_{0}\times 80$$

where *m*_0_ represents the mass of solid sodium silicate, g. *w*_0_ represents the dosage of Na_2_O, %. *W*_Na2O_ and *W*_SiO2_ represent the mass percentage of Na_2_O and SiO_2_ of solid sodium silicate, %. *M* represent the target modulus of the sodium silicate. *m*_NaOH_ represents the mass of solid NaOH granules, g.

#### 2.1.3. Other Materials

Tap water was used and its parameters met the requirements of the Chinese standard [27]. The ISO standard sand was selected to prepare SSG-mortar.

### 2.2. Test Methods

#### 2.2.1. Preparation of SSG-Mortar

The preparation process of SSG-mortar was as follows: Firstly, iron tailings sand, coal gangue powder, slag and standard sand were mixed thoroughly and transferred into a mixing pot. Secondly, solid sodium silicate, NaOH particles, and tap water were homogenized using a glass rod before being added to the mixing pot. Subsequently, the mixture was agitated at 140 ± 5 r/min for 30 s, followed by stirring at 285 ± 10 r/min for 30 s. A 90 s stirring pause was then implemented, after which the mixture was stirred again at 285 ± 10 r/min for 60 s to formulate the SSG-mortar.

#### 2.2.2. Compressive Strength Test

The compressive strength test of SSG-mortar was conducted in accordance with the Chinese Standard [28]. The SSG-sand mass ratio was 1:3, the ratio of water to binder was 0.45, and the mass of sand was 1350g. The solid mass of SSG-mortar included that of iron tailings sand, coal gangue powder, slag, NaOH granules and solid sodium silicate. The dimensions of SSG-mortar specimens were 40 mm in width, 40 mm in height and 160 mm in length. The specimens were subjected to curing under conditions of 20 ± 2 °C and no less than 95% of relative humidity (RH). The specimens were demolded following a 24 h curing period, and subsequently were cured for two additional durations (7 days and 28 days). Compressive strength tests were performed at a loading rate of 2400 ± 200 N/s. Each group of specimens was tested in triplicate, and the value of the compressive strength of SSG-mortar was computed in accordance with Equation (3).

(3) $$R_{c}=\frac{F_{c}}{A}$$

where *R_c_* denotes the compressive strength of the specimen, MPa. *F_c_* denotes the maximum load when the specimen is damaged, N. *A *denotes the pressurized area of the specimen, taken as 1600 mm^2^.

#### 2.2.3. Drying Shrinkage Test

Based on the Chinese Standard [29], a drying shrinkage test was conducted to evaluate the drying shrinkage property of SSG-mortar. The mass ratio of SSG-mortar to sand was set at 1:2, and the dimensions of SSG-mortar specimens were 25 mm in width, 25 mm in height and 280 mm in length. The curing and testing procedures were as follows: Firstly, the specimens, retained in their molds, were initially cured in a chamber controlled at 20 ± 1°C with RH ≥ 90% for 24 h. Following demolding, they were subjected to subsequent curing at 20 ± 3°C and 50 ± 4% RH for 4 h. Secondly, surface moisture of the specimens was then removed using a damp cloth, and their initial effective length was measured with a comparator (accuracy: 0.001 mm). Length measurements were performed periodically at 7-day intervals for ten consecutive cycles, with an additional measurement conducted at 120 days to evaluate the long-term shrinkage stability of the specimens. During testing, the pointer oscillation was required to be ≤ 0.02 mm, and readings were recorded to the precision of 0.001 mm. The shrinkage rate of specimens was calculated by Equation (4).

(4) $$\zeta t=\frac{L_{0}-L_{t}}{250}\times 100$$

where *ξ_t_* denotes the shrinkage rate of specimens at curing *t* days, %. *L_t_* denotes the specimen length at curing *t* days, mm. *L*_0_ denotes the reference length of specimens, mm.

#### 2.2.4. Freeze–Thaw Cycle Test

A freeze–thaw cycle test was performed by the Chinese Standard [30], utilizing the rapid freezing method to assess the frost resistance of SSG-mortar. The mass ratio of SSG-mortar to sand was set at 1:3. The dimensions of the SSG-mortar specimens were 40 mm in width, 40 mm in height and 160 mm in length. The curing and testing procedures were as follows: Firstly, the molded specimens were demolded and then subjected to water curing at 20 ± 2°C for 28 days. The specimens were then dried of surface moisture, after which their initial mass and compressive strength were tested. Secondly, the specimens were exposed to four different freeze–thaw cycle regimes, namely 25, 50, 75 and 100 cycles, and one freeze–thaw cycle condition was freezing at −17 ± 2°C for 2 h and then thawing at 8 ± 2°C for 1.5 h. After completing the designated freeze–thaw cycles, the surface moisture of the specimens was wiped, and then they were placed in an environment of 20 ± 2°C for 4 h to test the compressive strength and mass of the specimens. The testing procedure was terminated if the mass loss rate reached or exceeded 5%, or if the compressive strength loss rate reached or exceeded 25%. The mass loss rate and compressive strength loss rate of SSG-mortar were calculated respectively by Equations (5) and (6).

(5) $$\Delta W_{n}=\frac{W_{0}-W_{n}}{W_{0}}\times 100\%$$

where Δ*W_n_* denotes the mass loss rate of specimens after *n* freeze–thaw cycles, %. *W*_0_ and *W_n_* denote the mass of specimens before and after *n* freeze–thaw cycles respectively, g.

(6) $$\Delta f_{n}=\frac{f_{0}-f_{n}}{f_{0}}\times 100\%$$

where Δ*f_n_* denotes the compressive strength loss rate of specimens after *n* freeze–thaw cycles, %. *f*_0_ and *f_n_* denote the compressive strength of specimens before and after *n* freeze–thaw cycles respectively, MPa.

#### 2.2.5. Salt Erosion Test

Two types of salt erosion test, the dry–wet cycle test and the full immersion test, were conducted to research the salt erosion resistance of SSG-mortar according to the Chinese Standard [30]. The SSG-sand mass ratio was 1:3. The dimensions of SSG-mortar specimens were 40 mm in width, 40 mm in height and 160 mm in length. Four types of salt solutions were designed, including 3.5wt.%NaCl, 5wt.%Na_2_SO_4_, 5wt.%MgSO_4_ and a compound salt solution (a blend of 3.5wt.%NaCl, 5wt.%Na_2_SO_4_, 5wt.%MgSO_4_). The curing conditions of SSG-mortar specimens were 20 ± 2 °C and RH no less than 95% for 28 days. Full immersion testing was conducted with periods ranging from 5 to 30 days (namely, 5, 10, 15, 20, 25, and 30 days). After the specimens were immersed for the designed immersion time, the salt solution on the specimen’s surface was removed, and then stored in an environment with 23 ± 2°C and 70 ± 10% RH for 1 h until the surface of the specimens was dry. The salt solution’s dry–wet cycle condition involved immersing the specimens for 16 h, followed by wiping the surface solution and drying in an oven at 60 ± 2°C for 8 h, constituting one dry–wet cycle period. The test was carried out for 30 cycles in total, with a test being conducted every 10 cycles to calculate the mass loss rate and corrosion resistance coefficient of the SSG-mortar specimens using Equations (7)–(10).

(7) $$\Delta P_{m}=\frac{P_{a}-P_{m}}{P_{a}}\times 100\%$$

where Δ*P_m_* denotes the mass loss rate of specimens after immersion in the salt solution for *m* days, %. *P_a_* and *P_m_* denote the specimen mass prior to and following *m* day immersion in the salt solution respectively, g.

(8) $$\Delta k_{m}=\frac{k_{m}}{k_{a}}\times 100\%$$

where Δ*k_m_* denotes the erosion coefficient of specimens immersed in salt solution for *m* days. %. *k_m_* and *k*_a_ refer to the compressive strength of specimens after m days of immersion and after 28 days of curing (20 ± 2 °C, RH ≥ 95%), respectively, MPa.

(9) $${\Delta Q}_{n}=\frac{Q_{b}-Q_{n}}{Q_{b}}\times 100\%$$

where Δ*Q_n_* denotes the mass loss rate of specimens after *n* dry–wet cycles in the salt solution, %. *Q*_b_ and *Q_n_* denote the mass of specimens before and after *n* dry–wet cycles in the salt solution, respectively, g.

(10) $$\Delta h_{n}=\frac{h_{n}}{h_{b}}\times 100\%$$

where Δ*h_n_* denotes the erosion coefficient of specimens after* n* dry–wet cycles in the salt solution. *h_n_* denotes the compressive strength of specimens after *n* dry–wet cycles in the salt solution, MPa. *h*_b_ refers to the compressive strength exhibited by specimens upon completion of 30 days of curing, MPa.

#### 2.2.6. Scanning Electron Microscope Test

The central part of the SSG-mortar specimens was taken to conduct the scanning electron microscope (SEM) test. The hydration process of specimens was halted by anhydrous ethanol immersion, after which the samples were dried in a 50 °C vacuum drying oven. The fragments with relatively flat upper and lower surfaces were selected for gold spraying treatment. The microstructure of the SSG-mortar specimens is examined using a Quanta 3D FEG field-emission scanning electron microscope manufactured by FEI, Hillsboro, OR, United States.

#### 2.2.7. X-Ray Diffraction Test

XRD testing was carried out using a Smartlab 9kW X-ray diffractometer manufactured by Rigaku of Tokyo, Japan to analyze the phase changes in SSG-mortar. The scanning voltage and current were set at 40 kV and 40 mA, respectively, with a scanning angle range of 5° to 90° and a scanning speed of 2°/min.

## 3. Composition Optimization of SSG

### 3.1. Design Scheme and Results of RSM

Based on the RSM, a three-factor and three-level design (coded levels: −1, 0, 1) was conducted for SSG. The design variables were the dosage of coal gangue powder, slag, and Na_2_O [31,32,33], referring to Table 2. The total mass of iron tailing sand, coal gangue powder and slag was 100%. The 7-day and 28-day compressive strengths of SSG-mortar were selected as response indicators, and their results are presented in Table 3.

### 3.2. Analysis of Variance

Analysis of variance (ANOVA) was performed on the three response models—Linear, Quadratic, and 2FI—to select the most suitable model for predicting the compressive strength of SSG-mortar. The results of ANOVA are listed in Table 4. Based on the adjusted R^2^ (Adj-R^2^) values, the Quadratic model demonstrates superior goodness-of-fit compared to the Linear and 2FI models. The Adj-R^2^ values corresponding to 7-day and 28-day compressive strength are 0.9811 and 0.9662 for the Quadratic model, 0.6967 and 0.5726 for the Linear model, and are 0.9431 and 0.8713 for the 2FI model, respectively. Therefore, the Quadratic model indicates significantly higher regression accuracy than the Linear model and 2FI model, which is considered the most appropriate for establishing the relationship between SSG-mortar compressive strength and the dosages of coal gangue powder, slag and Na_2_O.

Taking a 95% confidence interval, ANOVA was conducted via the Quadratic model on the two response models corresponding to SSG-mortar’s 7-day compressive strength (Y_1_) and 28-day compressive strength (Y_2_), and the results are presented in Table 5 and Table 6.

As shown in Table 5, the *F*-value of the response model for the 7-day compressive strength of SSG-mortar is 93.51 (*p*-value <0.0001), and the *p*-value of Lack of Fit is 0.7022 (greater than 0.05), indicating that the response model for the 7-day compressive strength of SSG-mortar is statistically significant. The *p*-values for the coal gangue powder content (A), slag content (B) and Na_2_O content (C) are all less than 0.05, indicating that three factors have a significant effect on the 7-day compressive strength of SSG-mortar.

Furthermore, based on the *p*-value results, the order of influence of three factors on the 7-day compressive strength of SSG-mortar is: C > B > A. This is because, as the Na_2_O content increases, the elevated pH within the system promotes the leaching of [SiO_4_]^4−^ and [AlO_4_]^5−^ from the silico-aluminous raw materials, thereby accelerating the depolymerization-polymerization reaction process. However, an excessively high Na_2_O content (10%) leads to excessive initial alkalinity in the system, causing the depolymerization reaction to proceed too rapidly and results in an uneven distribution of the gel products [15]. The effect of slag content is secondary. Slag is rich in CaO (42.9%), which can rapidly release Ca^2+^ in an alkaline environment, which combines with the dissolved [SiO_4_]^4−^ and [AlO_4_]^5−^ to form C-S-H and C-A-S-H gels. These gel phases will form a skeletal network at an early stage, significantly enhancing the 7-day compressive strength of the SSG-mortar. The effect of coal gangue powder content is relatively minor. The reason may be that, although calcined and activated coal gangue powder possesses high pozzolanic activity, its CaO content is extremely low (1.55%). Early-stage hydration reactions are dominated by silicoaluminate gels, which have a slow nucleation rate, and thus make a limited contribution to the early strength of SSG-mortar [31].

Both interaction terms AB (coal gangue powder and slag) and BC (slag and Na_2_O content) exhibit significant effects (*p*-value <0.05). Regarding the AB interaction, the Ca^2+^ provided by the slag can undergo a synergistic reaction with the [AlO_4_]^5−^ released from the coal gangue powder, promoting the formation of C-A-S-H gel. This gel possesses a faster early-stage structural formation capacity compared to a single silicoaluminate gel. When the ratio of the two is appropriate, a high-density gel network will be formed to enhance the strength of the SSG-mortar. Regarding the BC interaction, the leaching of Ca^2+^ from the slag is closely related to the strongly alkaline environment provided by Na_2_O. When the Na_2_O content is moderate, it effectively promotes the dissociation of active components in the slag, enabling the synchronous release of Ca^2+^ and silicoaluminate clusters to form uniform and dense early-stage hydration products, thereby significantly enhancing the early strength of the SSG-mortar [18].

In addition, both the quadratic terms A^2^ and B^2^ reach the level of significance (*p*-value <0.05), indicating that there is an optimal range for the incorporation of both coal gangue powder and slag dosage.

As shown in Table 6, the incorporation of coal gangue powder (A), slag (B) and Na_2_O content (C) also have a significant effect (*p*-value <0.05) on the 28-day compressive strength of SSG-mortar.

The influence order is C > A > B, indicating a shift in the roles of the different components during the long-term hydration process. The Na_2_O content remains the most significant influencing factor. A moderate Na_2_O content maintains an appropriate alkalinity in the system during the later stages, promoting the continued dissociation of unreacted particles and further cross-linking of the gel network, whereas excessively high or low Na_2_O contents will limit the extent of the later-stage dissociation–condensation reactions of the silico-aluminous raw materials. The influence of coal gangue powder content surpasses that of slag content on the compressive strength at 28 days. The abundant Al_2_O_3_ (21.0%) in the coal gangue powder gradually exerts its effect during the later stages of hydration, and the C-A-S-H gel formed with the participation of Al^3+^ possesses a denser structure and superior long-term stability compared to pure C-S-H gel. The influence of slag content (B) relatively decreases on the compressive strength at 28 days. This is because the early hydration reaction of slag is rapid, and its Ca^2+^ is largely released and forms a gel network within 7 days, whilst the later stages primarily involve the restructuring and densification of the gel structure, resulting in a relatively slower contribution to strength development [34].

The interaction terms AB and BC remained significant on the 28 days compressive strength (*p*-value <0.05). During long-term curing, the AB interaction involves aluminum components gradually entering the silicate framework, where they balance charges with Na^+^ to form a C-(N)-A-S-H gel with higher polymerization. This structural evolution enhances the polymerization and strength of the gel network. Meanwhile, the BC interaction reflects the synergistic influence of Na_2_O and slag on the optimization of the microstructure in the later stages. An appropriate Na_2_O content allows the hydration reaction of the slag to be completed early, leading to the ordered rearrangement of gel products and the refinement of pores. Simultaneously, Na^+^ can enter the interlayer spaces of the gel via ion exchange, forming a C-(N)-A-S-H hybrid gel, thereby optimizing the pore structure and enhancing the density of the gel network.

The quadratic terms A^2^ and B^2^ remained significant on the 28 days compressive strength (*p*-value <0.05), further confirming that there is an optimal range for the incorporation of coal gangue powder and slag. Beyond the optimal dosage, an excess of coal gangue powder reduces the effective nucleation density of C-A-S-H in the gel products due to the dilution effect of active aluminum components, whilst an excess of slag, due to excessive Ca^2+^, tends to form high-calcium phases with poor stability, thereby weakening the 28-day compressive strength of SSG-mortar [32].

Based on the ANOVA results in Table 5 and Table 6, after removing non-significant terms, response models for the 7-day and 28-day compressive strengths of SSG-mortar were obtained, as shown in Equations (11) and (12). The coefficients of determination (R^2^) for the two models were 0.9918 and 0.9852, respectively, and the Adj-R^2^ values were 0.9811 and 0.9662, respectively. The negative effect of the Na_2_O content (C) was most significant, indicating that an excess of alkali activator led to the rapid formation of hydration gel products, resulting in a loose structure in SSG-mortar. The positive effect of the slag content (B) stems from its high calcium content, which promotes the nucleation of C-(A)-S-H gels. The negative effect of coal gangue powder content (A) is related to the slow release of its active aluminum components and the dilution of the reaction system. The coefficient of the interaction term AB and BC is negative, reflecting an antagonistic effect among coal gangue powder, slag and Na_2_O in their combination. In the quadratic terms, the negative coefficient of A^2^ confirms that there is an optimal range for coal gangue powder content. The positive coefficient of B^2^ indicates that, within the experimental range, no ‘over-blending’ inflection point has been reached for slag content, and the positive gain persists as the content increases.

(11) $$y_{7d}=32.35-0.9937A+1.03B-3.03C-2.38\mathrm{AB}-0.8\mathrm{BC}-0{.6313A}^{2}+0{.4812B}^{2}$$

(12) $$y_{28d}=38.09-1.10A+0.885B-3.14C-2.71\mathrm{AB}-1.41\mathrm{BC}-0{.9912A}^{2}+1{.16B}^{2}$$

**Figure 3:**
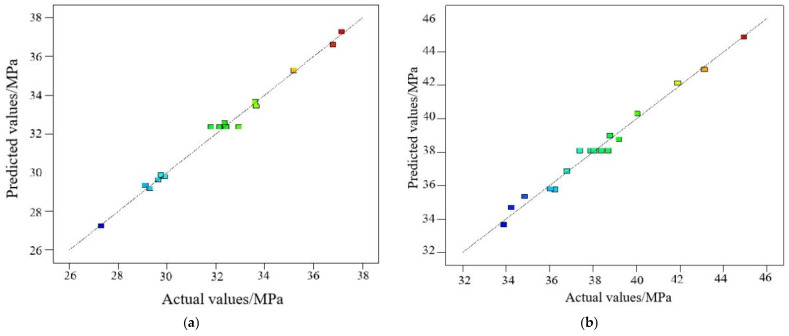
Predicted values vs. actual values of response models of SSG-mortar. (**a**) 7 days. (**b**) 28 days.

The consistency between predicted values and actual values of the compressive strength response models of SSG-mortar are compared, as illustrated in Figure 3. The predicted values of 7 days and 28 days align closely with the actual compressive strength values of SSG-mortar, displaying an approximately linear (45° slope) distribution. This suggests that the predicted values of two response models are highly consistent with the actual values.

**Figure 4:**
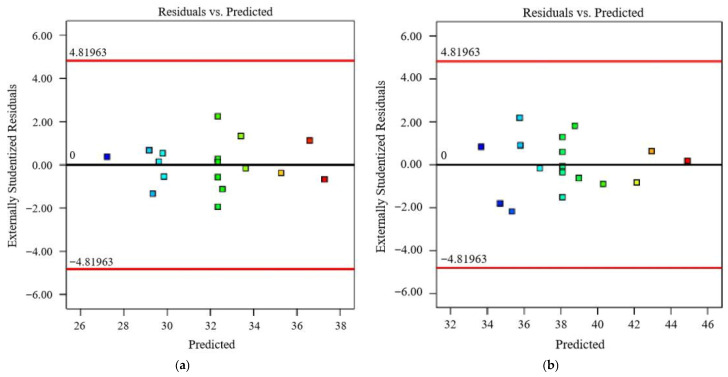
Externally studentized residuals of response models of SSG-mortar. (**a**) 7 days. (**b**) 28 days.

The normal distribution results of the predicted values of the SSG-mortar’s compressive strength response models at 7 days and 28 days are illustrated in Figure 4. All data points exhibit an approximately random distribution around the line y = 0, with over 90% of the points having absolute residuals within the range of −2 to +2. This indicates that the established compressive strength response models at 7 days and 28 days effectively capture the linear structure inherent in the data and maintains constant error variance. Consequently, it is feasible to conduct the component optimization design of SSG-mortar based on Equations (11) and (12).

### 3.3. Analysis of the Compressive Strength of SSG-Mortar

**Figure 5:**
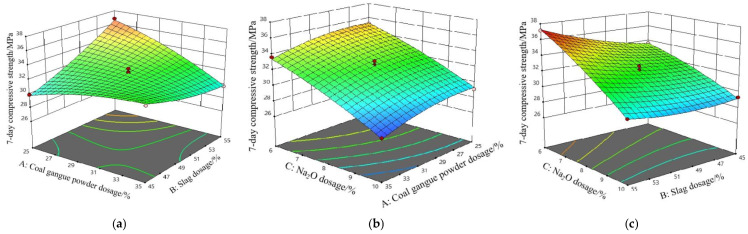
Results of 7-day compressive strength of SSG-mortar. (**a**) Coal gangue powder vs. slag. (**b**) Coal gangue powder vs. Na_2_O. (**c**) Na_2_O vs. slag.

**Figure 6:**
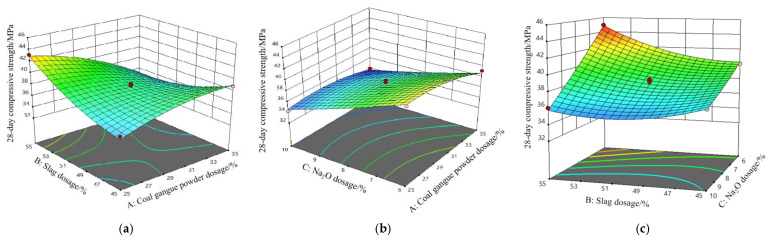
Results of 28-day compressive strength of SSG-mortar. (**a**) Coal gangue powder vs. slag. (**b**) Coal gangue powder vs. Na_2_O. (**c**) Na_2_O vs. slag.

The results of 7-day and 28-day compressive strength of SSG-mortar are shown in Figure 5 and Figure 6, respectively. As depicted in Figure 5a, with the increase in coal gangue powder and slag dosages, the 7-day compressive strength of SSG-mortar initially increases and then decreases. The maximum value of 7-day compressive strength (36.8 MPa) of SSG-mortar is achieved when the dosages of coal gangue powder and slag are 25% and 55%. In Figure 5b, as the dosages of Na_2_O and coal gangue powder decrease, the SSG-mortar’s compressive strength at 7 days increases gradually. The maximum value of 7-day compressive strength (35.2 MPa) of SSG-mortar is obtained when the dosages of Na_2_O and coal gangue powder are 6% and 25%. In Figure 5c, with the decrease in Na_2_O and slag dosages, the values of SSG-mortar’s compressive strength at 7 days increase. The maximum value of SSG-mortar’s compressive strength (37.2 MPa) at 7 days is observed when the dosage of slag and Na_2_O are 55% and 6%.

As illustrated in Figure 6a, the 28-day compressive strength values of SSG-mortar are originally enhanced and subsequently diminished with rising dosages of slag and coal gangue powder. Specifically, when the dosages of slag and coal gangue powder are 55% and 25%, the 28-day compressive strength values of SSG-mortar attain the maximum value (43.1 MPa). From Figure 6b, decreasing the dosages of Na_2_O and coal gangue powder, SSG-mortar’s compressive strength value at 28 days increases gradually. When the dosages of Na_2_O and coal gangue powder are 6% and 25%, the 28-day compressive strength value of SSG-mortar is 41.9 MPa. In addition, from Figure 6c, with the decrease in Na_2_O dosages and the increase in slag dosages, the 28-day compressive strength values of SSG-mortar raise gradually. When the slag dosage is 55% and the Na_2_O dosage is 6%, the 28-day compressive strength value of SSG-mortar reaches the maximum level (44.9 MPa).

There is an antagonistic interaction between coal gangue powder and slag. When the contents of coal gangue powder and slag increase simultaneously, the active components compete for the alkali activators. Furthermore, the limited interfacial compatibility between C-A-S-H gel and N-A-S-H gel led to uneven distribution of hydration products and reduction in the 7-day compressive strength of SSG-mortar. The negative impact of Na_2_O content is attributed to localized over-alkalization caused by an excess of alkali activators, resulting in the rapid formation of hydration gel products with a loose structure. Meanwhile, slag makes the positive contribution to the early strength of SSG-mortar under the suitable alkali activation conditions. The trend between 28-day compressive strength and SSG-mortar proportion is essentially consistent with 7-day compressive strength, which further confirms that the interactions between coal gangue powder, slag and Na_2_O have a significant impact on the compressive strength of SSG-mortar during both the short- and long-term curing stages [35].

### 3.4. Optimization and Verification of SSG

**Figure 7:**
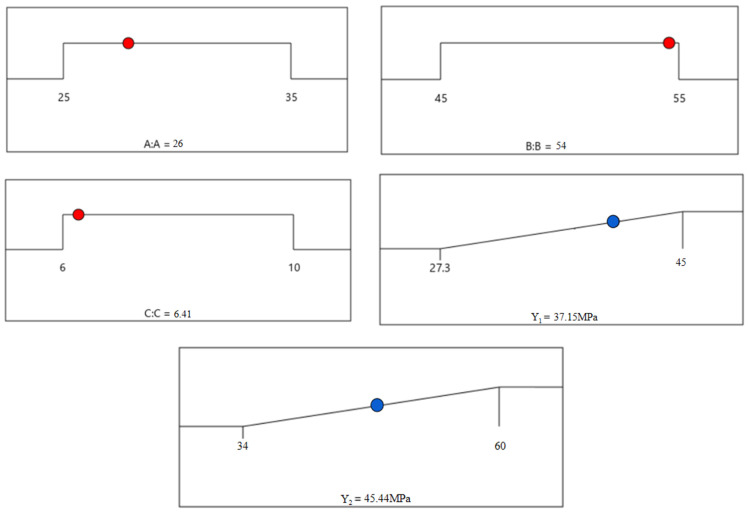
Analysis ramps of compressive strength of SSG-mortar.

Equations (11) and (12) are simultaneously computed to determine the optimal values of design variables. The 7-day and 28-day compressive strengths of SSG-mortar are designated as maximization targets. The analysis ramps are depicted in Figure 7. Each ramp is associated with a specific point that represents the intended aim for both the design variables and response variables. The meaning of the red dot in Figure 7 is the value of the ideal design parameters of SSG, and the blue dot is the predicted value of the compressive strength of SSG-mortar. According to Figure 7, the optimal design variables of SSG-mortar are 26% coal gangue powder, 54% slag, 20% iron tailings sand, and 6.41% Na_2_O (mixing with 6.23% solid sodium silicate and 6.23% solid NaOH granules).

Table 7 presents the optimal predicted values and the laboratory-measured values of the SSG-mortar. Laboratory tests were conducted in triplicate, and the deviation rate between the measured and predicted values was calculated via Equation (13) [36].

(13) $$Deviation\;rate(\%)=\frac{V_{Laboratory}-V_{Predicted}}{V_{Laboratory}}\times 100\%$$

It can be seen from Table 7 that the deviation rates corresponding to the two response variables are less than 2%, indicating that the laboratory test values are in good agreement with the predicted values. This finding confirms the feasibility of applying response surface methodology to optimize the SSG-mortar mix ratios for enhancing the mechanical performance of SSG-mortar.

## 4. Durability Performance of SSG-Mortar

### 4.1. Dry Shrinkage Performance

**Figure 8:**
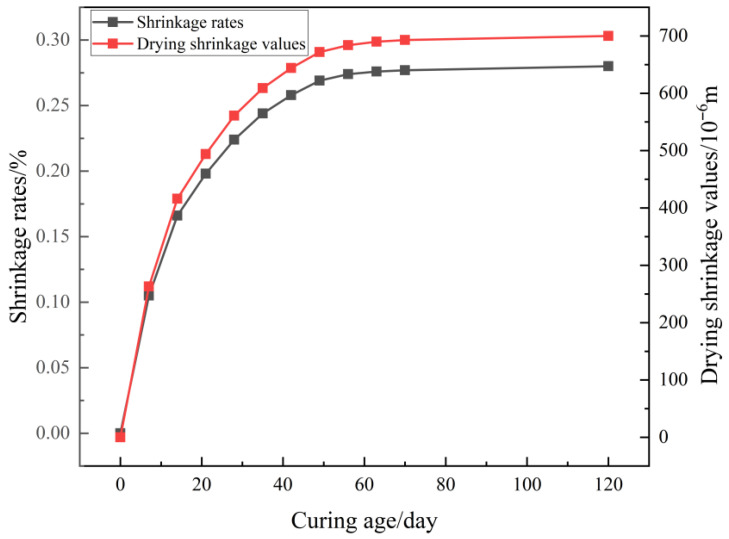
Results of drying shrinkage test for SSG-mortar.

The dry shrinkage test was conducted to research the dry shrinkage performance of SSG-mortar prepared by using the optimized composition of SSG. The test results are shown in Figure 8. As the curing age increases, the drying shrinkage value and shrinkage rate of SSG-mortar increase first and then stabilize. The drying shrinkage of SSG-mortar mainly occurs within the 28-day curing age, and the drying shrinkage value and shrinkage rate of SSG-mortar after curing 28 days are 5.61 × 10^−4^ m and 0.224%, respectively. Compared with the 28-day curing age, the drying shrinkage value and shrinkage rate of SSG-mortar after curing 56 days increase 21.9% and 22.3%, respectively. As the curing age increases from 56 days to 70 days, the drying shrinkage value and shrinkage rate of SSG-mortar rise 1.3% and 1.1%, respectively. Relative to the 70-day curing age, the drying shrinkage value and shrinkage rate of SSG-mortar specimens after curing 120 days increase 1.0% and 1.1%, respectively.

This phenomenon could be explained as follows: Before 28 days of curing, a substantial amount of free water exists in the SSG-mortar. At this stage, the internal humidity of the material was relatively high, and the free water evaporated rapidly through capillary action, leading to significant volume shrinkage. Between curing 28 and 56 days, as the hydration reaction of SSG-mortar continues, a large number of hydrated gels are produced in the mortar specimens and fill the structural pores. This process increases the specimen density and reduces the water migration channels, weakening the shrinkage trend of SSG-mortar. After the curing age exceeds 56 days, the active components in the SSG-mortar have fully reacted, and the gel network structure is basically finalized. Even if a small amount of water is lost, it is difficult to cause the volume changes in SSG-mortar [37].

### 4.2. Freeze Resistance Performance

**Figure 9:**
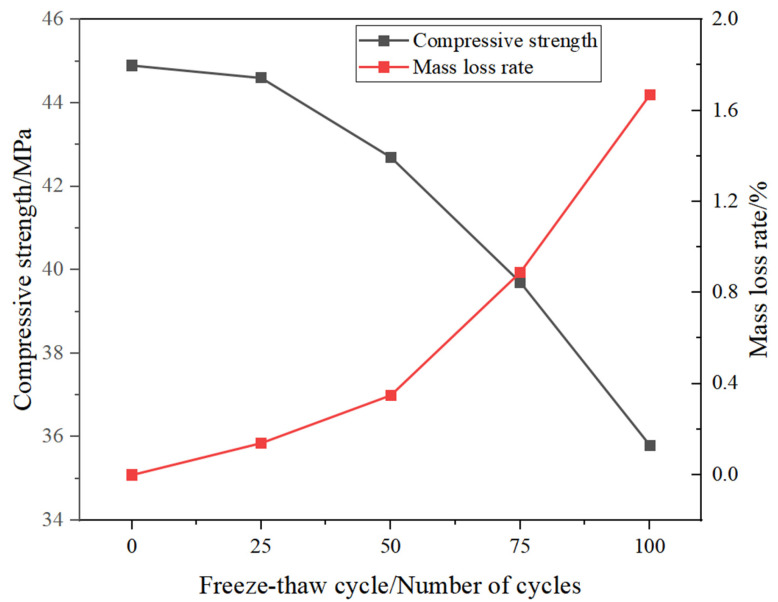
Results of freeze–thaw cycling test for SSG-mortar.

The results of the freeze–thaw cycle test of SSG-mortar are presented in Figure 9. As the cycle number increases, the mass loss rate of SSG-mortar rises gradually, whereas its compressive strength exhibits a progressive decline. In the initial phase of the freeze–thaw cycle test (0~25 cycles), the SSG-mortar demonstrates excellent spalling resistance, with a mass loss rate of merely 0.16%. As the number of cycles rises to 50, 75, and 100 times, the mass loss rate of SSG-mortar increases to 0.35%, 0.89%, and 1.67%, respectively.

Furthermore, the compressive strength loss rate of SSG-mortar increases significantly with the growing number of freeze–thaw cycles. In comparison to SSG-mortar that underwent no freeze–thaw cycles, the SSG-mortar’s compressive strength decreases by 0.6% (after 25 cycles), 4.9% (after 50 cycles), 11.6% (after 75 cycles), and 20.3% (after 100 cycles), respectively. The SSG-mortar’s compressive strength loss law is consistent with its accelerated mass loss phenomenon. Namely, the SSG-mortar specimens show relatively low mass loss rate and compressive strength loss rate at the early stage of the freeze–thaw cycle.

The reason may be that the micro-cracks accumulated within the mortar specimens gradually expand and merge during the freeze–thaw cycles, resulting in more water entering the interior of SSG-mortar. The frost pressure generated by the water freezing exceeds the bearing capacity of the internal gel network, leading to the local spalling of SSG-mortar [38].

### 4.3. Salt Erosion Resistance Performance

**Figure 10:**
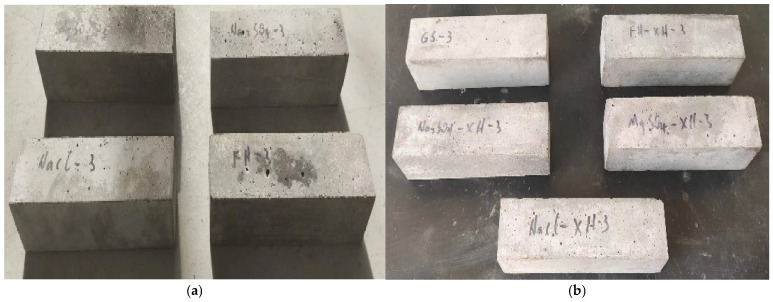
Appearance of SSG-mortar specimens. (**a**) Immersion in salt solution for 30 days. (**b**) 30 dry–wet cycles in salt solution.

The appearance of SSG-mortar specimens after 30 days of full immersion and 30 dry–wet cycles in salt solutions is represented in Figure 10. Under two salt corrosion test conditions, no significant deterioration is observed on the surface of SSG-mortar specimens. The specimen surfaces are intact without cracks, and the edges and corners remain clear. Especially in the composite salt solution, only a slight darkening of the surface color is observed on the specimens, while no surface spalling or chalking—phenomena that typically occur on cement-based mortar in salt corrosion environments—are found. This indicates that SSG-mortar has a good salt corrosion resistance.

**Figure 11:**
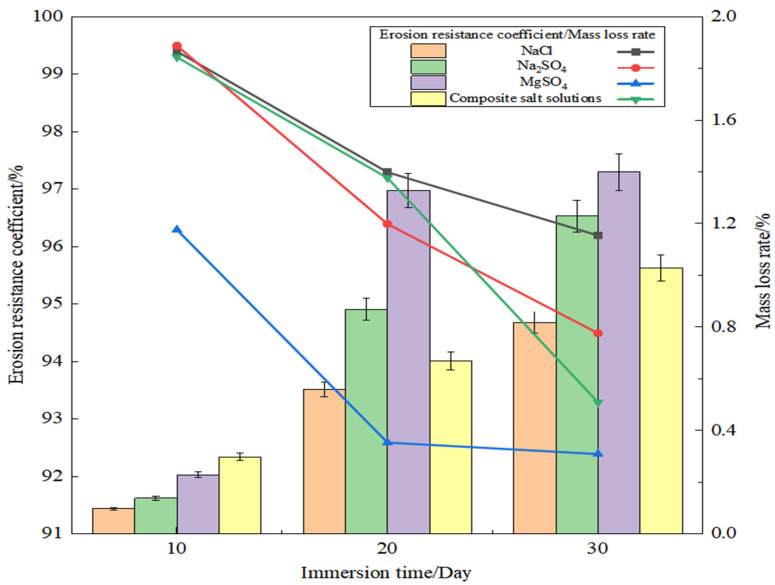
Results of SSG-mortar after immersion in different salt solutions.

Figure 11 illustrates the mass loss rate and erosion resistance coefficient of SSG-mortar under various salt erosion conditions. As the immersion time increases, the mass loss rate of SSG-mortar specimens exhibits a gradual upward trend. In comparison to the 10-day immersion, the mass loss rates of SSG-mortar specimens after 20-day immersion in NaCl, Na_2_SO_4_, MgSO_4_, and composite salt solutions increase by 4.60, 5.21, 4.78, and 1.23 times, respectively. For the 30-day immersion, the corresponding increments are 7.20, 7.79, 5.09, and 2.43 times. When the immersion time is 10 days, the composite salt solution causes the highest mass loss rate of SSG-mortar at 0.3%, followed by the MgSO_4_ solution, which induces a mass loss rate of 0.23%. After 20-day and 30-day immersion, the four salt solutions demonstrate a uniform trend in affecting the SSG-mortar’s mass loss.

Compared with the 10-day immersion, the erosion resistance coefficients of SSG-mortar specimens immersed in MgSO_4_, NaCl, Na_2_SO_4_, and composite salt solutions for 20 days decreased by 3.8%, 2.1%, 3.1%, and 2.1%, respectively. For 30-day immersion, the corresponding reductions were 4.0%, 3.3%, 5.0%, and 6.0%, resulting in a final erosion resistance coefficient of 92.4%, 96.2%, 94.5%, and 93.3% for the four types of salt solutions, correspondingly.

This result indicated that the MgSO_4_ solution possesses a strongest erosive capacity toward the SSG-mortar. Although the NaCl solution causes slight surface dissolution due to Cl^−^ penetration, its lowest mass loss rate suggests that Cl^−^ has a relatively weak destructive effect on the aluminosilicate network structure. Notably, the salt corrosion effect of the composite salt solution is weaker than that of single Na_2_SO_4_ or MgSO_4_ solutions. The reason may be that the increased variety of ions in the composite salt solution leads to a relatively lower concentration of the highly corrosive SO_4_^2−^ ions [39]. The reduced surface contact between SO_4_^2−^ and SSG-mortar specimens consequently results in a lower mass loss rate compared to that in single salt solution. Compared with immersion in MgSO_4_ solution, SSG-mortar specimens in Na_2_SO_4_ solution exhibit a smaller mass loss rate. The reason is that Mg^2+^ has a stronger erosion effect on the C-(A)-S-H gel. Mg^2+^ undergoes an ion exchange reaction with Ca^2+^ in the C-(A)-S-H gel, thereby further disrupting the internal cementitious structure of SSG-mortar [40].

**Figure 12:**
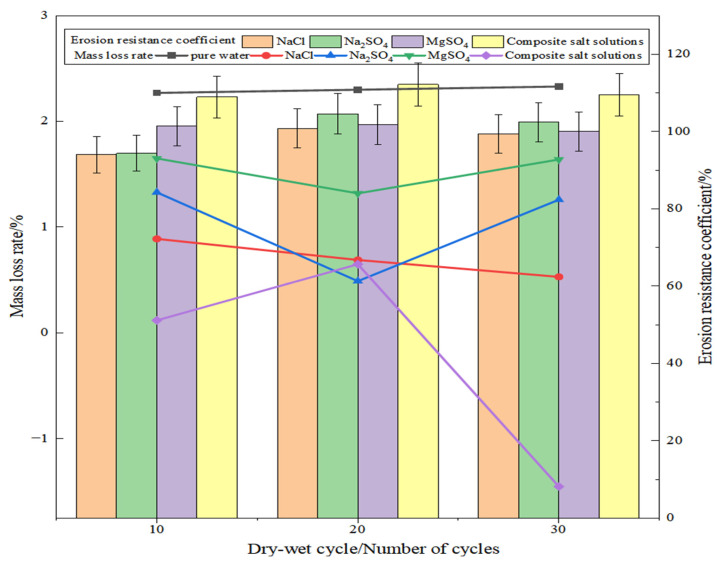
Results of SSG-mortar after dry–wet cycle erosion in different salt solutions.

Figure 12 illustrates the erosion resistance coefficient and mass loss rate of SSG-mortar after undergoing dry–wet cycles in different salt solutions. Compared with pure water, the mass loss rate values of SSG-mortar after dry–wet cycles in different salt solutions are all relatively small. The reason is that the ions in the salt solutions could react with the silicon–aluminum raw materials in the geopolymer to form ettringite and other compounds. Moreover, the dry–wet cycles lead to an increased presence of salt crystals within the geopolymer pores, consequently filling the internal pores and decelerating the erosion caused by the salt solution [41]. Compared with the four types of salt solution conditions, as the progression of dry–wet cycles increases, the SSG-mortar’s mass loss rate values in the Na_2_SO_4_ and MgSO_4_ solution conditions exhibit a tendency of preliminary decrease then subsequent rise, and the change law of the SSG-mortar’s mass loss rate subjected to Na_2_SO_4_ solution salt erosion is more significant. In contrast, the mass loss rate of SSG-mortar specimens under the NaCl solution salt erosion shows a gradually decreasing trend. Compared with 10 dry–wet cycles, the mass loss rate values of SSG-mortar specimens after 20 and 30 dry–wet cycles decrease by 1.3% and 2.6%, respectively. In addition, under the composite salt solution condition, with the increase in the number of dry–wet cycles, the mass loss rate value of SSG-mortar specimens shows a trend of first increasing and then decreasing, and after 30 dry–wet cycles, the mass loss rate value of SSG-mortar specimens is −1.5%, which is due to the fact that there are more salt ions in the composite salt solution, and more salt crystals are filled in the SSG-mortar pores.

In addition, the erosion resistance coefficients of SSG-mortar specimens in different salt solutions exhibit a tendency of preliminary rise then subsequent decline as the number of dry–wet cycles increases. This tendency is particularly pronounced under the dry–wet action of the Na_2_SO_4_ solution. The SSG-mortar has the highest erosion resistance coefficient after undergoing dry–wet cycles under composite salt solution conditions, reaching 112.2% after 20 cycles. In contrast, the SSG-mortar has the lowest corrosion resistance coefficient in the NaCl solution, with 100.8% after 20 dry–wet cycles. Compared with 10 dry–wet cycles, the SSG-mortar’s erosion resistance coefficient value increases by 7.2%, 10.8%, 0.4%, and 3.0% in NaCl, Na_2_SO_4_, MgSO_4_, and composite salt solutions after 20 dry–wet cycles. The coefficients of SSG-mortar after 30 dry–wet cycles show respective increases of 5.7%, 8.5%, −1.4% and 0.6% compared with the 10 dry–wet cycles.

The main reason may be that, during the early phase of the cycles, salt particles infiltrated the internal pores of SSG-mortar, which not only optimized the internal microstructure of the specimens but also enhanced its compressive strength. As dry–wet cycles proceeded, the negative effect of the salt solution on the erosion of SSG-mortar progressively increased, and the erosion damage exceeded the pore-filling reinforcement effect, leading to the reduction in the compressive strength [42].

## 5. Reaction Mechanisms

### 5.1. XRD Tests

**Figure 13:**
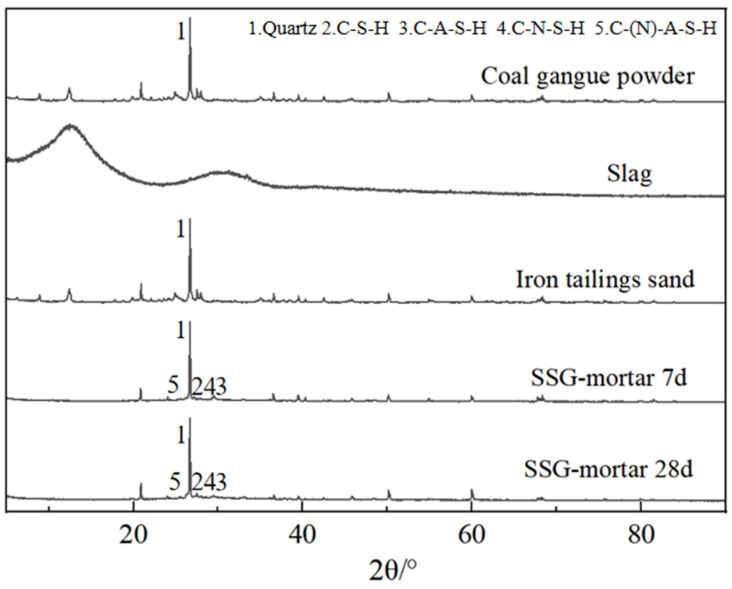
XRD patterns of the raw materials and SSG-mortar.

To investigate the effect of curing time on SSG-mortar prepared with the optimal mix design, an XRD test was conducted on iron tailings sand, coal gangue powder, slag, and SSG-mortar cured for 7 and 28 days, respectively, as shown in Figure 13. The XRD patterns of coal gangue powder and iron tailings sand exhibit sharp crystalline peaks, primarily corresponding to inert crystalline phases such as quartz (2θ ≈ 26.6°). Slag primarily exhibits a typical ‘doughnut peak’, indicating an amorphous glassy structure (2θ ≈ 20–35°) with high potential cementitious activity. Both the 7-day and 28-day SSG-mortars retain the characteristic quartz peak, whilst the amorphous ‘doughnut-shaped peak’ gradually broadens with increasing age. Furthermore, at 28 days, the intensity of some weak crystalline peaks increases compared to the 7-day samples. This is due to the depolymerization–polymerization reaction of silico-aluminous raw materials in an alkaline environment, resulting in the formation of C-S-H gel (2θ ≈ 32°) and a small amount of amorphous N-A-S-H, C-A-S-H and C-(N)-A-S-H gel (2θ ≈ 25–35°). The extent of the reaction is lower in the early stages, and changes in the crystalline phase are not pronounced. However, as the curing time increases, more active components participate in the polymerization, leading to the extensive formation and micro-crystallization of amorphous gel, which broadens the amorphous peak and makes the weak crystalline peaks more distinct. This indicates that, as the curing age increases, the cementitious products within the SSG-mortar continue to develop and the structure gradually becomes denser [43].

### 5.2. SEM Text

**Figure 14:**
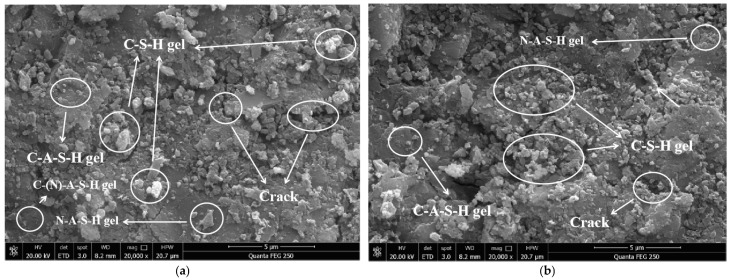
Microstructures of SSG-mortar after curing different times. (**a**) 7-day. (**b**) 28-day.

The microstructures of the SSG-mortar specimens after curing 7 and 28 days are shown in Figure 14. In the alkali-activated system, silico-aluminous raw materials undergo depolymerization–polymerization reactions under alkaline conditions. OH^−^ breaks the Si-O-Si and Si-O-Al bonds in the raw materials, leaching out [SiO_4_]^4−^, [AlO_4_]^5−^ and ions such as Ca^2+^ and Na^+^. As the slag in the raw materials provides a rich source of calcium, whilst coal gangue powder and iron tailings sand provide silicon and aluminum components, the coexistence of Ca, Si, Al and Na in the system results in the reaction products exhibiting diverse characteristics. On the one hand, Ca^2+^ combines with [SiO_4_]^4−^ and [AlO_4_]^5−^ to form C-A-S-H gel. On the other hand, Na^+^ participates in the gel structure to form N-A-S-H gel.

In actual reaction processes, these two often coexist in the form of a composite C-(N)-A-S-H gel, forming a three-dimensional network framework. As the curing period is extended from 7 days to 28 days, the ongoing dissolution–condensation reaction within the system leads to the continuous generation and accumulation of gel products. The originally dispersed gels gradually coalesce into a unified whole, and the network structure becomes increasingly dense. At the same time, some of the loose structures formed in the early stages undergo rearrangement and reconstruction in the alkaline environment, resulting in tighter bonding in the interfacial transition zone. This microstructural evolution, characterized by the synergistic filling of the gel network and unreacted particles, is the fundamental reason for the continuous increase in the compressive strength of SSG-mortar with age [44].

**Figure 15:**
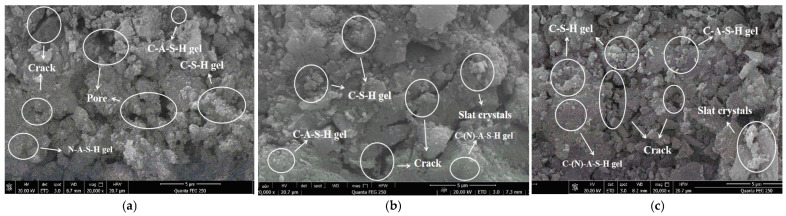
Microstructures of SSG-mortar after durability test. (**a**) Freeze–thaw cycles. (**b**) Erosion by NaCl solution. (**c**) Dry–wet cycles.

The microstructures of SSG-mortar following freeze–thaw cycles and salt erosion are shown in Figure 15. In Figure 15a, the surface of the SSG-mortar exhibits distinct cracks following freeze–thaw cycles, and the number of voids has increased. This is due to the water within the pores expanding in volume upon freezing, generating frost heave pressure that repeatedly compresses the surrounding gel network. Under the influence of multiple freeze–thaw cycles, the cumulative effect of this frost heave pressure causes microcracks to propagate in the weaker regions of the gel network, eventually interconnecting to form macroscopic cracks. Simultaneously, the interfacial bonding between some unreacted particles and the gel is weakened, leading to a decline in structural integrity and, consequently, a reduction in compressive strength [43].

In Figure 15b, after 30 days of exposure to NaCl solution, a small amount of salt crystals appears on the surface of the SSG-mortar. Compared with the specimen cured under standard conditions for 28 days, the surface exhibits larger cracks and the hydrated gel products are more dispersed. This is primarily due to the penetration of Cl^−^, which disrupts the ionic equilibrium within the gel structure. Some Na^+^ ions combine with Cl^−^ to form crystalline precipitates on the surface, whilst the leaching of Ca^2+^ causes localized decalcification of the C-A-S-H and C-(N)-A-S-H composite gels. This reduces the stability of the gel matrix, leading to the loosening and cracking of the originally dense network structure. Compared with Figure 15b, Figure 15c shows that, after 30 wet–dry cycles in NaCl solution, the SSG-mortar exhibits increased surface cracking and a looser structure. Under wet–dry cycling conditions, the repeated infiltration and evaporation of the salt solution intensify the processes of ion migration and recrystallization within the gel. During the drying phase, salt supersaturation within the pores leads to crystallization pressure. During the wetting phase, the re-penetration of the solution further leaches out ions such as Ca^2+^ and Na^+^, causing the gel network to deteriorate continuously. The alternating action of these two processes causes microcracks to continuously expand into interconnected cracks, and the bond between the gel and unreacted particles is gradually lost, ultimately leading to a significant decrease in the compressive strength of the SSG-mortar [45].

## 6. Conclusions

1. Composition optimization of solid sodium silicate-activated solid waste-based geopolymer can be effectively achieved using the response surface methodology. The optimal compositions of geopolymer are 20% iron tailings sand, 26% coal gangue powder, 54% slag, and 6.41% Na_2_O content (namely, mixed by 6.23% solid sodium silicate and 6.23% NaOH granules).
2. Solid sodium silicate-activated solid waste-based geopolymer exhibits a better resistance to shrinkage and salt corrosion. Across all tested salt solutions, the mass loss rate of geopolymer mortar remains below 2.4%, while its erosion resistance coefficient stays above 92%. As the curing age increases, the drying shrinkage value of geopolymer mortar rises, while the growth rate gradually diminishes. Following freeze–thaw cycle treatment, the rate of compressive strength loss and rate of mass loss for geopolymer mortar are below 18.7% and 1.67%, respectively.
3. The improvement in mechanical performance of geopolymer mortar can be attributed to the development of a dense gel network structure by hydration products. Additionally, hydration products and solid waste raw materials occupy the pores within the geopolymer mortar, enhancing its structural compactness.

## Figures and Tables

**Table 1:** Chemical compositions of geopolymer precursors.

Experimental Material	Mass Percentage/%							Activity Index/%
	SiO_2_	CaO	Al_2_O_3_	MgO	Na_2_O	Fe_2_O_3_	K_2_O	
Iron tailings sand	68.3	6.57	4.59	4.72	0.96	12.2	1.39	81.50
Coal gangue powder	61.5	1.55	21.0	2.55	1.52	5.55	4.4	89.28
Slag	27.3	42.9	12.4	11.0	0.66	0.65	0.55	-

**Table 2:** Design factors and levels for SSG.

Levels	Coal Gangue Powder Dosage (A)/%	Slag Dosage (B)/%	Na_2_O Dosage (C)/%
−1	25	45	6
0	30	50	8
1	35	55	10

**Table 3:** Response surface methodology design and results for SSG-mortar.

Number	A/%	B/%	C/%	Y_1_ (7-Day Compressive Strength)/MPa	Y_2_ (28-Day Compressive Strength)/MPa
1	30	55	6	37.15	44.95
2	30	45	6	33.60	40.05
3	25	50	6	35.20	41.90
4	35	50	6	33.65	39.20
5	25	55	8	36.80	43.13
6	25	45	8	29.90	36.25
7	35	55	8	29.75	34.85
8	30	50	8	32.45	38.70
9	30	50	8	32.15	38.40
10	30	50	8	31.80	38.05
11	30	50	8	32.40	37.40
12	30	50	8	32.95	37.90
13	35	45	8	32.35	38.80
14	30	45	10	29.30	36.80
15	35	50	10	27.30	33.90
16	30	55	10	29.65	36.05
17	25	50	10	29.10	34.25

**Table 4:** ANOVA results for the compressive strength regression model of SSG-mortar.

Model Types	7-Day Compressive Strength			28-Day Compressive Strength		
	*F*-Value	*p*-Value	Adj-R^2^	*F*-Value	*p*-Value	Adj-R^2^
Linear	17.81	0.0069	0.6967	22.34	0.0045	0.5726
Quadratic	0.5000	0.7022	0.9811	1.58	0.3264	0.9662
2FI	3.28	0.1349	0.9431	7.25	0.0379	0.8713

**Table 5:** ANOVA results for the 7-day compressive strength response model of SSG-mortar.

Category	Sum of Squares	Degree of Freedom	Mean Square	*F*-Value	*p*-Value	Significance
Model	118.20	9	13.13	93.51	<0.0001	Significant
A	7.90	1	7.90	56.25	0.0001	Significant
B	8.41	1	8.41	59.84	0.0001	Significant
C	73.51	1	73.51	523.39	<0.0001	Significant
AB	22.56	1	22.56	160.65	<0.0001	Significant
AC	0.02	1	0.02	0.11	0.7485	Not significant
BC	2.56	1	2.56	18.23	0.0037	Significant
A^2^	1.68	1	1.68	11.95	0.0106	Significant
B^2^	0.98	1	0.98	6.94	0.0337	Significant
C^2^	0.69	1	0.69	4.95	0.0615	Not significant
Residual	0.98	7	0.14			
Lack of Fit	0.27	3	0.09	0.50	0.7022	Not significant
Pure Error	0.72	4	0.18			
Cor Total	119.19	16				

**Table 6:** ANOVA results for the 28-day compressive strength response model of SSG-mortar.

Category	Sum of Squares	Degree of Freedom	Mean Square	*F*-Value	*p*-Value	Significance
Model	142.85	9	15.87	51.78	<0.0001	Significant
A	9.64	1	9.64	31.43	0.0008	Significant
B	6.27	1	6.27	20.44	0.0027	Significant
C	78.75	1	78.75	256.90	<0.0001	Significant
AB	29.32	1	29.32	95.65	<0.0001	Significant
AC	1.38	1	1.38	4.50	0.0715	Not significant
BC	7.98	1	7.98	26.03	0.0014	Significant
A^2^	4.14	1	4.14	13.50	0.0079	Significant
B^2^	5.65	1	5.65	18.44	0.0036	Significant
C^2^	0.19	1	0.19	0.63	0.4543	Not significant
Residual	2.15	7	0.31			
Lack of Fit	1.16	3	0.39	1.58	0.3264	Not significant
Pure Error	0.98	4	0.25			
Cor Total	145.00	16				

**Table 7:** Validation results of compressive strength of SSG-mortar.

Response Indicators	Unit	Predicted Values	Laboratory Values	Deviation Rate/%
A	%	26	26	
B	%	54	54	
C	%	6.41	6.41	
Iron tailings sand	%	20	20	
7-day compressive strength (Y_1_)	MPa	37.15	36.82	−0.90
28-day compressive strength (Y_2_)	MPa	45.44	44.90	−1.20

## Data Availability

The original contributions presented in this study are included in the article. Further inquiries can be directed to the corresponding author.
